# Recurrent Implantation Failure Is More Frequently Seen in Female Patients with Poor Prognosis

**Published:** 2012-06-19

**Authors:** Pelin Ocal, Tayfur Cift, Berk Bulut, Eray Balcan, Ismail Cepni, Begum Aydogan, Tulay İrez

**Affiliations:** 1 Department of Obstetrics and Gynecology, Istanbul University, Cerrahpaşa Medical School, Istanbul, Turkey; 2 Ministry of Health of Turkey, Malkara State Hospital, Tekirdağ, Turkey

**Keywords:** *In Vitro* Fertilization, Implantation, Recurrent Implantation Failure

## Abstract

**Background:**

This study evaluated the characteristics and results of patients who suffer from recurrent implantation failure (RIF).

**Materials and Methods:**

In this cross sectional study, a total of 2183 cases who were evaluated retrospectively at the Istanbul University Cerrahpasa Medical Faculty, Department of Obstetrics and Gyneacology, IVF unit between 2000-2007. According to the data gathered, we included 1822 cases in this study. We compared 185 patients with RIF to 1637 women without RIF.

**Results:**

Pregnancy was achieved by 589 couples out of 1822 (32%). The implantation rate was 10%, which declined to 5.8% after the fourth attempt. In the RIF group, patients’ mean age was higher and there were more overweight women, the duration of fertility was longer, day 3 follicle stimulation hormone (FSH) levels and the total gonadotropin dose administered were higher, mean level of Estradiol (E2) on the human chorionic gonadotropin (hCG) day was lower, and the mean level of progesterone on the hCG day was elevated compared to the non-RIF group. Although the comparison of MII oocyte number was not significant, the mean number of fertilized oocytes was found to be significant in favor of the non-RIF group. The endometrial thicknesses were found to be similar for both groups. Comparison of sperm motility and morphology were statistically significant in favor of the RIF group.

**Conclusion:**

In our study, we have found that the group with RIF were comprised of patients with poor prognosis who were older, overweight, had a longer infertility duration, a higher FSH level, and needed more gonadotropin doses in controlled ovarian hyperstimulation (COH). Sperm motility and morphology were better in the RIF group compared to the non-RIF group, and multiple pregnancy rates were lower in RIF patients.

## Introduction

Recurrent implantation failure (RIF) is defined as the lack of any pregnancy in three consecutive *in vitro* fertilization/ intracytoplasmic sperm injections- embryo transfer(IVF/ICSI-ET) cycles or ten good-quality embryo transfers (1-3). Results of recent studies reveal that it is a financial and legal obligation to restrict the number of embryos transferred (4-6). Currently it is preferable to transfer only one embryo or two embryos (4-6). In Turkey, in concordance with the guidelines of the Ministry of Health General Directorate of Maternal and Infant Health and Family Planning, the transfer of a single embryo at initial two attempts, excluding special circumstances, has been legalized. Formerly, couples were diagnosed RIF if implantation failed after three consecutive IVF/ICSI-ET cycles or after the transfer of ten good quality embryos. However, single embryo transfers according to new legal measures make it a necessity to reconsider the definition of RIF.

In recent years, great advances have been achieved in the treatment of infertile couples. However despite these advances, there are still some infertile couples who suffer from RIF. Probable underlying etiologies for RIF are aneuploidy of embryos, uterine cavity abnormalities, diminished endometrial response, and insufficiencies in transfer techniques (2). These factors result in decreased pregnancy rates even at successful IVF centers, and RIF remains a problem, of both economical and psychological aspects for couples.

Our aim was to investigate the characteristics of our patients who suffer from RIF and to discuss the management protocols in view of the literature.

## Materials and Methods

In this cross sectional study, patients who underwent IVF/ICSI cycles at İstanbul University Cerrahpasa School of Medicine, Department of Obstetrics and Gynecology, IVF Unit from January 2000-January 2007 were retrospectively reviewed to locate those patients diagnosed with RIF. A total of 1822 cases out of 2183 were included in the study. Of these, 185 RIF patients were compared to 1637 patients who did not have RIF. Patients with cycle cancellation and no oocyte during oocyte pick up or men without sperm at TESE were all excluded from the study. The inclusion criteria was: age limit of 42 years, basal follicle stimulation hormone (FSH) (day 3) level of <20 mIU/mL, and normal gynecological ultrasound and cervical smear. All patients were given a written informed consent. The local Institutional Ethics Committee approved the study.

All patients received the GnRH agonist leuprolide acetate (1 mg/day sc Lucrin®, Abbott- France Pharmaceuticals, France) beginning on the 21^st^ day the of previous cycle (long protocol) or the first day of the cycle (short protocol). Leuprolide acetate was reduced to 0.5 mg/day and gonadotropin 150-450 IU (Menogon®, Ferring, Istanbul; Gonal F®, Merck Serono, Istanbul; or Puregon®, Schering Plough, Istanbul, Turkey) were initiated on the third day of menstruation according to age, body mass index (BMI), basal FSH value, and prior ovulation induction trials.

Controlled ovarian hyperstimulation (COH) was monitored by transvaginal sonography, and the gonadotropin dose was adjusted according to follicle size and number. When three or more follicles reached >18 mm, we administered 10000 IU of human chorionic gonadotropin (hCG, Pregnyl®, Schering Plough, Istanbul, Turkey) for ovulation induction.

Oocyte aspiration was performed transvaginally, 35-36 hours after administration of the hCG injection. During the oocyte pick-up procedure, sedative anesthetics or local anesthesia was used. Sequential medium was used for embryo culture and transfer. Embryos were selected for transfer by pronuclei scoring, cleavage rate, fragmentation, and blastomere equivalence scoring. Assisted hatching was applied to embryos which had thick zona pellucida layers. Quality of embryos and age of the patients were the main factors in determining the number of embryos to be transferred. Hard manipulations, bleeding from cervix during the transfer procedure, or the use of a tenaculum were considered "difficult transfer".

The luteal phase was supported by progesterone (200 mg, Progynex®, Koçak, Istanbul, or Crinone gel® 8%, Merck Serono, Istanbul) administered vaginally three times daily or 100 mg progesterone IM injections daily (Progynex ® ampule, Koçak, Istanbul). In appropriate cases, embryos were followed until the blastocyst phase and transfer was performed at that time. Clinical pregnancy was defined as the detection of a gestational sac on the ultrasound. Implantation rate was defined as the number of gestational sacs over the number of embryos transferred. Pregnancy rate was defined as the number of pregnancies with visible fetal heart activity on ultrasound examination over the number of transferred embryos.

### Statistical analysis

Results were expressed as mean ± SD, frequency, and percentages. Analyses were performed by Unistat 5.1 software. Categorical characteristics of patients were compared with the chi square test. Independent Samples t-test and Mann Whitney U tests were used for comparison of numeric variables. P<0.05 was considered statistically significant.

## Results

In our study, 589 couples achieved pregnancy out of 1822 (32%). Implantation rates were as follows: 10% (first attempt; n=1424); 9.6% (second attempt; n=435); 11% (third attempt; n=201); 5.8% (fourth attempt; n=91); 2% (fifth attempt; n=41), and 6% for >5 attempts (n=75). Success rates diminished significantly after the third attempt.

According to age, implantation and pregnancy rates were 10% and 29.5% under 35 years; implantation rate was 7% and pregnancy rate was 25.7% between 35-39 years while implantation rate was 2% and pregnancy rate was 12.8% over 40 years.

Subjects had the following diagnoses: tuboperitoneal factor (314), male factor (1320), polycystic ovary syndrome (PCOS, 115), unexplained infertility (50), hypogonadotropic hypogonadism (14), uterine factor (4), and 5 had endometriosis (Table 1).

**Table 1:** Etiology of the infertile patients


Infertility etiology	Number (n)

**Tubo-peritoneal factor**	314
**Male factor**	1320
**Polycystic ovary syndrome (PCOS)**	115
**Unexplained infertility**	50
**Hypogonadotropic hypogonadism**	14
**Uterine factor**	4
**Endometriosis**	5
**Total**	1822


The group characteristics and results have been given in table 2. Non-RIF patients constitute group I, which included 1637 cases. Group II comprised 185 cases of RIF. The mean age was 32.48 ± 5.24 years in group I and 35.93 ± 4.76 years in group II (p<0.0001). Mean duration of infertility was 8.25 ± 5.05 years in group I and 11.04 ± 5.30 years in group II (p<0.0001). The mean weight of subjects in group I was 65.92 ± 10.85 kg and 69.85 ± 11.52 kg in group II (p<0.003). Mean values for waist circumferences were 82.88 ± 10.89 cm in group I and 88.12 ± 12.89 cm in group II (p<0.016).

**Table 2:** Group’s characteristics and treatment features


	Group I (≤3 attempts)	Group II (>3 attempts)	P value

**Age (Y)**	32.48± 5.24	35.93± 4.76	0.0001
**Infertility duration (Y)**	8.25 ± 5.05	11.04 ± 5.30	0.0001
**Weight (Kg)**	65.92± 10.85	69.85± 11.52	0.003
**Waist (cm)**	82.88± 10.12	88.12± 12.89	0.016
**3^r^^d^ day FSH (IU/ml)**	7.45 ± 3.86	8.95 ± 6.55	0.0001
**Total gonadotropin ampules**	29.85± 13.93	37.93± 15.30	0.0001
**Endometrium (mm)**	10.27 ± 2.45	11.33 ± 1.28	ns
**Total gonadotropin dose (IU)**	2391.45 ± 1209	2989.02 ± 1262	0.0001
**E2 on hCG day (pg/ml)**	2001.95 ± 1617.75	1621.47 ± 1184.36	0.003
**Progesterone on hCG day (ng/ml)**	0.96 ± 0.63	3.37 ± 1.48	0.001
**MII oocytes**	4.1 ± 3.5	3.9 ± 3.9	ns
**Transferred embryos**	3.49 ± 1.49	3.27 ± 1.65	0.045
**Sperm motility (%)**	66.00 ± 23.36	72.32 ± 20.15	0.001
**Sperm morphology (Kruger’s criteria) (%)**	3.00 ± 2.19	3.81 ± 2.27	0.0001


ns; Not significant.

Day 3 FSH values were 7.45 ± 3.86 IU/ml in group I and 8.95 ± 6.55 IU/ml in group II (p<0.0001). The total number of 75 IU gonadotropin ampules administered was 29.85 ± 13.93 in group I and 37.93 ± 15.30 in group II (p<0.0001). Total gonadotropin dose was 2392.45 ± 1209.49 IU in group I and 2989 ± 1262.37 IU in group II (p<0.0001).

Estradiol (E2) values on hCG day were 2001.95 ± 1617.75 pg/ml in the first group and 1621.47 ± 1184.36 pg/ml in the second group (p<0.003). The mean value of serum progesterone level on the hCG day was 0.96 ± 0.63 ng/ml in group I and 3.37 ± 1.48 ng/ml in group II (p<0.001).

The mean number of MII oocytes were 4.1 ± 3.5 in group I and 3.9 ± 3.9 in group II; there was no statistical significance between groups. The mean endometrial thicknesses were 10.27 ± 2.45 mm in group I and 11.33 ± 1.28 mm in group II, which was not statistically significant.

The mean number of fertilized oocytes were 4.78 ± 3.05 in the non-RIF group and 4.13 ± 2.95 in the RIF group (p<0.006). The number of transferred embryos was 3.49 ± 1.4 in the non-RIF and 3.27 ± 1.65 in the RIF group (p<0.045).

Blastocyst transfers were done in 18 patients in group I and 7 patients in group II. The mean number of transferred blastocysts were 2 ± 1.32 in group I and 3.14 ± 0.69 in group II (p<0.043). Assisted hatching was applied to 19 patients in group I and 8 patients in group II.

According to Kruger’s criteria, the sperm parameter morphology was 3.81 ± 2.27 % (group II) vs. 3 ± 2.19 % (group I) and motility was 72.32 ± 20.15% (group II) vs. 66 ± 23.36% (group I) (p<0.001).The values were found to be significantly better in the RİF group.

The groups were also compared for singleton and multiple pregnancies. In the first group, rates for singleton pregnancies were 13.5%, twin were 5.4%, and triple were 1.9%, while they were 8.5% (single), 3.5% (twin), and 0.5% (triple) in the second group. Multiple pregnancy rates were lower in the RIF group compared to the non-RIF group.

Embryo transfer was classified into easy and difficult transfers. In group I the transfer was easy in 91.6% of cases, and in group II it was easy in 92.9% of cases. The transfer technique was not significant between the groups. Ultrasonography was used in 52.3% of the transfers in group I and 44% of the transfers in group II; there was no significant difference.

**Table 3:** Group’s pregnancy rates


Pregnancy rates	Group I (≤3 attempts)	Group II (>3 attempts)	P value

**Singleton pregnancy (%)**	13.5	8.5	0.041
**Twin pregnancy (%)**	5.4	3.5	0.041


## Discussion

Recurrent IVF failure continues to be an important problem and a distressing condition for couples. Despite recent advances in medical technology, implantation rates still remain low. Our implantation rate was reported as 10% for the first attempt and diminished significantly after the third attempt. As noted in the literature, implantation and pregnancy rates decrease after the fourth attempt (7).

In our study, we found that the RIF group consisted of women with poor prognoses. In contrast, sperm morphology and motility were better in that group. Severe male factor was treated successfully in the first attempts, as they were probably married to normal fertile women.

Women in the RIF group had advanced mean age, higher day 3 FSH levels, longer infertility durations, and a higher mean weight when compared to non-RIF women. These differences were all statistically significant and can be defined as poor prognoses factors for women who underwent IVF.

It has long been known that with increasing age there is a decline in natural fecundity and pregnanacy rates. Along with the decrease in follicle number, the oocyte quality also diminishes (8).

In recent decades, numerous reports regarding the outcome of ART treatment have confirmed that the probability of a live birth decreases distinctly after the age of 35 years (9).

Walsh et al. stated that couples with RIF had poor prognosis and the pregnancy rates decreased further over the age of 35 (10).

In our study the mean age of the RIF group was 35.93 years, which was older than the non-RIF group. Maheshwari et al. have concluded that older women were more likely to have a diagnosis of unexplained infertility compared with those who were younger. They also reported that the duration of infertility was more widespread in older women, which was compatible with our findings (11).

Both increasing age and basal FSH were significantly associated with reduced numbers of oocytes collected, oocytes fertilized, and embryos transferred. Markers of ovarian reserve, day 3 FSH, inhibin B and E2, anti-müllerian hormon (AMH), antral follicle count (AFC) are particularly predictive and useful in guiding the choice of the optimal protocol for ART. However, no tests have been absolutely predictive of a successful outcome. Today there is no technology that can predict the IVF outcome or estimate the RIF group (12).

In our study we found a statistically significant difference for weight between the two groups in favor of the non-RIF group. The published data regarding the effect of body mass index (BMI) on IVF cycles is varied. Some studies highlighted a state of gonadotropin resistance in obese women, which lead to higher gonadotropin requirement for COH (13). Many studies indicated lower implantation and pregnancy rates and higher miscarriage rates (14). Nichols et al. and Wang et al. both have reported reduced conception rates in overweight women undergoing IVF (15, 16). Other studies did not show any adverse effects of obesity on endometrial thickness, hormone levels, oocyte number and quality, implantation, and pregnancy rates (17, 18).

In our study the numbers of metaphase II oocytes obtained were similar in both groups, but in the RIF group we used a higher amount of gonadotropin and a lower mean level of E2 on hCG day was calculated.

Kably Ambe et al. have shown that E2 levels on hCG day is not an influential factor on pregnancy rates, especially in older patients (19). In contrast, Orvieto et al. have evaluated the influence of the ratios of E2 to the number of follicles >14 mm on the day of hCG administration (E2/follicle) by comparing the two different protocols. They reported that within the antagonist group higher pregnancy rates were observed when comparing those with an E2/oocyte ratio of 100-200 pg/ml to others who had an E2/oocyte ratio <100 pg/ml or >200 pg/ml (20).

In our study the serum progesterone level on the hCG day was higher in the RIF group due to early luteinization. Early luteinization incidence varies, ranging between 5% and 30% in IVF patients. It may adversely affect the clinical outcome and could be related to diminished ovarian reserve. It is not necessarily a LH-de pendent event and is observed mostly in women of advanced age (21).

Concurrent to our study, Ozturk Turhan et al. have reported that in the group whose progesterone levels were higher than 1.5 ng/ml on the hCG day, mature oocytes, fertilization, and cleavage rates were significantly lower (p<0.05) (22).

Early luteinization leads to more post-mature oocytes at oocyte pick-up and higher progesterone levels disturb endometrial maturation and integrity. Furthermore, fewer oocytes are fertilized and go under cleavage. As a result, the progesterone level has been shown to be higher in the RIF group and the mean number of transferred oocytes was lower compared to the non- RIF group.

Sperm count and motility were better in the RIF group, which lead us to conclude that our RIF patients constituted abundant, poor responder women and accordingly we needed to obtain better quality oocytes and prevent premature luteinization.

We measured endometrial thickness on the hCG day and did not find any difference. Richter et al. stated that thicker endometrium increased clinical pregnancy, continuing pregnancy and live birth rates independent from age and embryo quality (23). In another study, pregnancy rates dropped at endometrial thicknesses under 7 mm, but the authors recommended embryo transfer because pregnancies were obtained (24).

In our study, more embryos were transferred in patients without RIF. They also had a greater number of embryos available for transfer, which was statistically significant (p<0.024). In IVF procedures, the embryo number to be transferred is increased when the patient has adverse prognostic factors such as higher age, poor embryo quality, and RIF. Fewer embryos are transferred in couples with secondary infertility that already have healthy children. Although it is the usual practice to transfer three embryos in Turkey, in couples who obviously have a better prognosis single embryo transfer is the treatment of choice. Frequently up to three embryos could be transferred in the presence of advanced maternal age and/or poor embryo quality. However, even in the presence of an obviously positive prognosis, more than half of the physicians prefer to transfer three embryos, and the percentage of doctors choosing single embryo transfer has remained below 15% (25).

The guidelines published in 2006 emphasized that embryo transfers yielded more successful outcomes if they were performed at the blastocyst stage in IVF cycles in order to reduce and prevent multiple pregnancies. It has been determined essential to transfer one or two embryos for those under 35 years old, a maximum of three embryos for those between 35-37, three embryos between 38-39, and four embryos for those over 39 years of age (26). In Turkey, the most recent regulations that came into effect in 2010 stipulated application of single embryo transfer in infertile women under the age of 35 in the first two attempts (27). This implicated the need for a new definition of RIF.

In order to increase implantation rate, more blastocysts were formed and transferred in the RIF group. Transferring the embryos in blastocyst stage resulted in higher implantation and live birth rates in the RIF group (28, 29). Margalioth et al. demonstrated that blastocyst transfer increased implantation rates in patients with RIF (2). In our clinical practice we also prefer to transfer more blastocyst stage embryos in RIF patients.

The practice of assisted hatching was more frequent in the non-RIF group. Sallam et al. concluded that assisted hatching increased the rates of ongoing pregnancy, implantation and pregnany (30). Cochrane data indicated that assisted hatching increased clinical pregnancy rates, but there was insufficient evidence about the effect on live birth rates (31). Currently there is inadequate evidence to recommend routine assisted hatching.

As recommended in the literature, in our unit blastocyst transfer is preferred in appropriate patients, assisted hatching is used for thick zona pellucidas, embryo quality is evaluated by embryo scoring systems, and embryos of the highest quality are transferred, in order to have a better implantation and pregnancy rate.

In the literature, ulitrasound-guided embryo transfer was associated with increased rates of clinical, ongoing, and live pregnancy rates compared with the transfers made without ultrasonography guidance (32). In our study, the groups had no difference in terms of transfer technique and transfer difficulty.

## Conclusion

In our study we found that the group with RIF was composed of poor prognosis patients who were older, overweight, had a longer infertility duration, an elevated FSH level, and needed to use more gonadotropins in COH.

Sperm motility and morphology were better in the RIF group compared to the non-RIF group and multiple pregnancy rates were lower in RIF patients. In such patients, the RIF probability must be taken into account and an appropriate treatment must be made individually.
